# 

**Published:** 1907-04

**Authors:** 


### BOOKS RECEIVED.

A Textbook of the Practice of Medicine. For Students and Practitioners. By Hobart Amory Hare, M.D., B.Sc., Professor of Therapeutics and Materia Medica in the Jefferson Medical College of Philadelphia; Physician to the Jefferson Medical College Hospital; Laureate of the Royal Academy of Medicine in Belgium and of the Medical Society of London. Author of A Text-Book of Practical Therapeutics; A Text-Book of Practical Diagnosis, etc. In one very handsome octavo volume of 1120 pages, with 131 engravings and 11 full-page plates in colors and monochrome. Second edition, revised and enlarged. Lea Brothers & Co., Philadelphia and New York. 1907. (Cloth, $5.00; leather, $6.00; half morocco, $6.50, net prices).

---

Textbook of Psychiatry. A Psychological Study of Insanity for Practitioners and Students. By E. Mendel, M.D., A. O. Professor in the University of Berlin. Authorised translation. Edited and enlarged by William C. Krauss, M.D., Buffalo, N. Y., President Board of Managers Buffalo State Hospital for Insane; Medical Superintendent Providence Retreat for Insane; Neurologist to Buffalo General, Erie County, German, Emergency Hospitals, etc.; Member of the American Neurological Association. 311 pages. Crown Octavo. Philadelphia: F. A. Davis Company. (Price, Extra Cloth, $2.00 net).

---

Medical Diagnosis. A Manual of Clinical Methods for practitioners and students. Fifth edition, greatly enlarged and revised to date, by J. J. Graham Brown, M.D., F.R.C.P.E., F.R.S.E., Assistant Physician, Royal Infirmary of Edinburgh, and W. T. Ritchie, M.D., F.R.C.P.E., F.R.S.E., Clinical Assistant Pathologist, Royal Infirmary of Edinburgh. 12 mo., pp. 524. With 200 illustrations and 8 full page plates in black and white and in color. New York: Imperial Publishing Company. 1907. (Price, $3.00).

---

Progressive Medicine, Vol. I, March, 1907. A Quarterly Digest of Advances, Discoveries and Improvements in the Medical and Surgical Sciences. Edited by Hobart Amory Hare, M.D., Professor of Therapeutics and Materia Medica in the Jefferson Medical College of Philadelphia. Octavo, 280 pages, with illustrations. Lea Brothers & Co., Philadelphia and New York. (Per annum, in four cloth-bound volumes, $9.00; in paper binding, $6.00, carriage paid to any address).

---

Paraffin in Surgery. A critical and clinical study by Wm. H. Luckett, M.D., Attending Surgeon, Harlem Hospital, Surgeon to the Mt. Sinai Hospital Dispensary of New York and Frank I. Horne, M.D., Formerly Assistant Surgeon, Mt. Sinai Hospital Dispensary. 12 mo.; 38 illustrations; 118 pages. Surgery Publishing Co., 92 William Street, N. Y. City. (Cloth, $2.00).

---

Medical Epitome Series. Diseases of the Nose and Throat. By J. B. Ferguson, M.D., Instructor in Diseases of the Nose and Throat in the N. Y. Post-Graduate Medical School. 12mo., 243 pages, with 114 engravings. Edited by Victor C. Pedersen, M.D., Lecturer in Surgery at the New York Polyclinic Medical School and Hospital. Philadelphia and New York: Lea Brothers & Co. (Price, $1.00 net).

---

Psychology applied to Medicine. Introductory Studies by David H. Wells, M.D., Lecturer on Mental Physiology and Assistant in

Ophthalmology, Boston University Medical School. 12 mo. pp. 155. Philadelphia: F. A. Davis Co. 1907. (Price, $1.50 net).

---

Students Aid Series. Aids to Dental Surgery. By Arthur S. Underwood, M.R.C.S., L.D.S., Eng. And Douglas Gabell, M.R.C.S., L.R.C.P., Lond., L.D.S., Eng. Second edition. 126 pp. New York: William Wood & Co. (Price, $1.00).

---

Aids to Medical Diagnosis. By Arthur Whiting, M.D., M.R.C.P., Physician to the Tottenham Hospital; Lecturer in and Dean of the Northeast London Post-Graduate College. 152 pages. New York: William Wood & Co. (Price, $1.00).

---

Principles and Application of Local Treatment in Diseases of the Skin. By L. Duncan Bulkley, A.M., M.D., Physician to the New York Skin and Cancer Hospital. Small 8vo., 142 pages. New York: Rebman Co. (Price, $1.00).

---

Aids to the Diagnosis and Treatment of Diseases of Children. By John McCaw, M.D., Physician to the Belfast Hospital for Sick Children. Third edition. 383 pages. New York: William Wood & Co. (Price, $1.25).

---

The Johns Hopkins Hospital Reports. Vol. XII. Studies in Urological Surgery. Vol. XIV. Studies on Hypertrophy and Cancer of the Prostate. Baltimore: The Johns Hopkins Press. 1906.

---

Surgery of the Genitourinary Organs. By J. W. S. Gouley, M.D., Small 8vo. pp. 531. New York: Rebman Co. (Price, $3.00).

